# The Novel Fusion Proteins, GnRH-p53 and GnRHIII-p53, Expression and Their Anti-Tumor Effect

**DOI:** 10.1371/journal.pone.0079384

**Published:** 2013-11-04

**Authors:** Peiyuan Jia, Yu Zhao, Shaoping Wu, Junhua Wu, Shan Gao, Ying Tong, Yuxia Wang

**Affiliations:** 1 Beijing Institute of Pharmacology and Toxicology, Beijing, China; 2 Beijing Center of Disease Prevention and Control, Beijing, China; Rush University Medical Center, United States of America

## Abstract

p53, one of the most well studied tumor suppressor factor, is responsible to a variety of damage owing to the induction of apoptosis and cell cycle arrest in the tumor cells. More than 50% of human tumors contain mutation or deletion of p53. Gonadotrophin-releasing hormone (GnRH), as the ligand of Gonadotrophin-releasing hormone receptor (GnRH-R), was used to deliver p53 into tumor cells. The p53 fusion proteins GnRH-p53 and GnRH iii-p53 were expressed and their targeted anti-tumor effects were determined. GnRH mediates its fusion proteins transformation into cancer cells. The intracellular delivery of p53 fusion proteins exerted the inhibition of the growth of H1299 cells in vitro and the reduction of tumor volume in vivo. Their anti-tumor effect was functioned by the apoptosis and cell cycle arrest induced by p53. Hence, the fusion protein could be a novel protein drug for anti-tumor therapy.

## Introduction

As a 10-amino acid regulator of reproduction, gonadotrophin-releasing hormone (GnRH) is released by neurons in the hypothalamus, and is transported via the hypothalamo-hypophyseal portal circulation to the anterior pituitary. It triggers gonadotropin release for luteinizing hormone and follicle-stimulating hormone, which in turn stimulate the gonads for steroid production. GnRH transmits its signal via specific receptors that belong to the large group of G-protein coupled receptors (GPCRs) [1]. Gonadotrophin-releasing hormone receptor (GnRH-R) was found to be expressed in normal human reproductive tissues (e.g. breast, ovary, endometrial, prostate, pancreas, colon, lung and liver) and tumors derived from these tissues [2-5]. Numerous studies have provided evidence, indicating the suppress effect of GnRH and GnRH-a (GnRH analogue) in these tumor cells proliferation. The multiple events of GnRH could be associated with the divergence of signaling pathways that are activated by GnRH-R.

Lamprey gonadotropin-releasing hormone III (lGnRH III) is the third isoform of GnRH isolated from the sea lamprey (*Petromyzon marinus*) [6]. As a weak agonist of the endocrine activity of GnRH-R, GnRH III has been identified to recognize the binding sites on human cancer cells, and then inhibit the cells growth within micromolar concentrations. GnRH-III significantly suppressed the growth of human cancer cells expressing GnRH receptors [7-10]. lGnRH-III was applied as a targeting moiety to deliver anticancer agents into tumor cells [11]. lGnRH-III and its analogs represent a new outlook for researching trends of the application of GnRH compounds for chemotherapy.

In addition to the application of GnRH and its analogues, oriented tumor inhibition research of GnRH conjugated with the drug, toxin or nanoparticle via the GnRH receptor recognition effect has made significant progress. Moreover, abundant evidences indicate that GnRH is capable for mediating transmembrane delivery with different molecule cargos like polypeptides, proteins and nanoparticles via recognizing its receptor. It is revealed a new strategy for developing anticancer reagents associated with GnRH [4,12-14]. 

The tumor suppressor p53 controls numerous downstream targets that can result in variable outcomes, including apoptosis, transient growth arrest, and sustained growth arrest or senescence. The expression of p53 gene is induced by DNA damage to prevent gene amplification and preserve genetic stability. This protein induces G0/1 phase arrest via binding on the specific DNA sequence and activating its downstream genes such as p21 [15]. P53 functions to eliminate and inhibit the proliferation of abnormal cells, thereby preventing neoplastic development. Cell canceration and dropout of growth caused by genetic deletion and mutation are the main mechanisms during tumorigenesis. Epidemiological data demonstrated that p53 is mutated in more than half of all human tumors; most of the p53 mutant points are located in its DNA-binding core domain [16]. Therefore p53 has been an appealing target for new anticancer therapeutic strategies. The utility of p53 protein as a biological agent is limited by poor permeability and low targeting-delivery. 

In this study we investigated the cell-permeable p53 fusion proteins conjugated with GnRH or GnRH III for targeting therapy on the tumors containing p53 gene mutation or deficient. 

## Materials and Methods

### The construction of GnRH-p53 and GnRH III-p53

GnRH (GeneID: 409109), GnRH III(GeneID: 360141) and p53 gene (cDNA in GenBank , Accession No. BC003596) was fused and cloned into the pET28a vector. p53 cDNA was prepared from human embryonic total RNA by following the cDNA synthesis protocol (Promega, USA) and amplified by PCR [17]. GnRH and GnRH III fragments were introduced with primers GnRH P1, GnRH P2, GnRH III P1 and GnRH III P2 by PCR using pET28a-p53 as a template (Table 1). The PCR product was purified and extracted using Gel recycling Kit (TianGen CO., LTD. Beijing) and digested with NheI and XhoI. The fragment was cloned into pET28a (Cat. No.69864-3, Novagen, 5369bp), sequenced, and matched with the p53 gene.

**Table 1:** Primers of PCR for amplification of GnRH and GnRH III.

**Primers**	**Sequence**
GnRH P1	5’CTAGCATGGAGCACTGGTCCTATGGACTGCGCCCTGGAA3’
GnRH P2	5’AGCTTTCCAGGGCGCAGTCCATAGGACCAGTGCTCCATG3’
GnRH III P1	5’CTAGCATGGAGCACTGGTCCCACGACTGGAAGCCTGGAA3’
GnRH III P2	5’AGCTTTCCAGGCTTCCAGTCGTGGGACCAGTGCTCCATG3’

GnRH P1 and GnRH III P1were sense sequences; GnRH P2 and GnRH III P2 were antisense sequences. The cohesive terminis of restriction enzyme Nhe I and Hind III were underlined.

GnRH: pGlu-His-Trp-Ser-Tyr-Gly-Leu-Arg-Pro-Gly-NH_2_

GnRH III:pGlu-His-Trp-Ser-His-Asp-Trp-Lys-Pro-Gly-NH_2_

Primary structures of GnRH and GnRH III. The variable region of the GnRH family of peptides is underlined.

### Expression and Purification of p53 Fusion Proteins

The fusion proteins, GnRH-p53 and GnRH III-p53, were expressed in *E coli*. BL21(DE3) pLysS (TianGen BIOTHEC CO., LTD. Beijing). As the control protein, wild type p53 (p53), was also prepared. The bacteria were cultured in LB medium containing 50 μg/ml kanamycin for 8 h, and then added into 2×YT medium at 1:100 (v/v) dilutions. Then the bacteria were grown for 4h at 37 °C and 1 mmol/L IPTG was added for the final 6h for induction [18]. After centrifugation at 5000g for 10 min precipitations were resuspended with PBS, and ultrasound on ice for 25 cycles with 10 s on and 45 s off. The homogenates were centrifuged at 12,000g for 10 min at 4°C. The pellets were washed with washing buffer (2 M urea, 100 mM NaCl, 50 mM PBS, pH 8.0) overnight at 4 °C. The washed pellets were dissolved in 8M urea, and loaded onto a Ni-NTA column at 4°C purified by affinity chromatography. Proteins loading in bag filter of 50 kDa MWCO (Genestar CO., LTD. Shanghai.) were dialyzed in renature buffer (0.1 mM GSH, 0.01 mM GSSG, 0.1 mM EDTA and 50 mM PBS, pH 7.5) to remove urea and allow protein refolding, and finally dialyzed against PBS. Protein concentration was measured according to Lorry [19] using bovine serum albumin as standard. Approximately 6 mg protein was recovered per liter of bacterial culture. Protein was stored in 50 mM PBS at -80°C.

Purity of the GnRH-p53 and GnRH III-p53 was assessed by electrophoresis using 12% polyacrylamide gels with SDS Laemmli buffer followed by Commassie staining (Simply Blue SafeStain, Invitrogen). The bands migrating at 54 KDa were identified. Western blotting was accomplished by transfer of proteins following SDS-PAGE onto nitrocellulose with transfer buffer (25 mM Tris, 190 mM glycine, 20% MeOH, pH 8.3) through semi-dry transfer chamber, and exposure of the monoclonal antibody against human p53 (DO-1, Santa Cruz, America) at 1:1,000 dilution overnight at 4° C. After washing with TBST (50 mM Tris–HCl, pH7.5, 150 mM NaCl, 1% Tween-20) and addition of secondary antibody, goat anti-mouse IgG conjugated to horseradish peroxidase (Jackson, America) with 1:1,000 dilution at 37°C for 45 min, the band was developed using ECL (Applygen Technologies Inc., Beijing, China).

### MTT assay

H1299 cells (non-small cell lung cancer cells , p53 deficient cells) were added into 96-well culture plates (Costar 3359, America) at a density of 5×10^4^ cells per well. Cells were treated for 48 h with purified p53, GnRH-p53 and GnRH III-p53 dissolved in RPMI-1640 culture media. The 5 mg/ml solution of MTT(Sigma, America) in 10 mM PBS was added to the culture medium, in a volume of 20 μl per well. After 4 h, the culture medium was removed and replaced with 200 μl DMSO per well. 96-well culture plates were shaken for 20 min at room temperature to dissolve the particles completely. The values of optical density (OD) were measured at 540 nm on a micro-ELISA reader (Labsystem Multiskan MCC/340, Finland). Cell growth inhibition rate was calculated via OD_540_ for cells treated with fusion protein or with culture media alone (as control).

The GnRH analogues Leuprorelin (GnRH-R agonist, Sigma) and G662 (GnRH-R antagonist, synthesized by our institute) were also added into H1299 cell with MTT assay (described above).

### Immunocytochemistry and Immunofluorescence analysis

According to the method described by Yu Zhao, et al [20] H1299 cells were seeded into six-well culture plates at a density of 5×10^5^ cells per well and cultured 24h. The p53, GnRH-p53 and GnRH III-p53 proteins were added to a final concentration of 40 μg/ml and PBS also added as control. After incubated for 2 h, the culture medium was removed and the cells were washed for 5 min three times with cold PBS. Cells were fixed with 95% ethanol for 2 h at 4°C and washed with PBS for 5 min three times. Cells were blocked with 10% goat serum for 30 min at room temperature and incubated with 1:500 dilution of the mouse against human p53 monoclonal antibody (DO-1, Santa Cruz) at 4°C overnight. Immunostaining was carried out by Histostain^TM^-Plus Kits (Zymed, America) according to the manufacturer’s instructions. An avidin/biotin horseradish peroxidase system was used with DAB to localize the site of immunoreactive antigen and photographed.

To assess the permeability across membranes of p53 fusion proteins, H1299 cells incubated with p53, GnRH-p53 and GnRH III-p53 (40 μg/ml) or PBS for 4h were analyzed by immunofluorescence. Samples were blocked with 10% goat serum for 30 min at room temperature. The cells were incubated with monoclonal antibody DO-1 and rabbit against human GnRHR polyconal antibody (Boster CO., LTD. Wuhan) at 4 °C overnight. TRITC-Conjugated AffiniPure Goat Anti-mouse IgG and FITC-Conjugated AffiniPure Goat Anti- rabbit IgG (Zhongshan Golden Bridge CO., LTD. Beijing) were used as second antibodies and added into six-well culture plates at 37°C for 1h. After washing for 5 times with cold PBS, the cells in wells were incubated with Hoechst 33342 at room temperature for 10 minutes in order to dye the cell nuclei. Cells were washed with PBS for 5 min three times and viewed with a fluorescence microscope.

### Apoptosis of cells

The flow cytometry was used to analyze the apoptosis in the proteins treated H1299 cells. H1299 cells were seeded into six-well culture plates at a density of 5×10^5^ cells per well and cultured for 24h. The p53, GnRH-p53 and GnRH III-p53 proteins or RPMI1640 cell culture media were added and incubated with H1299 cells for 12h, 24h, and 48h, respectively. H1299 cell line was incubated with Annexin V-FITC/PI and the apoptosis of H1299 cell was monitored by flow cytometry. 

### Animal experiments

This study was carried out in strict accordance with the guidelines of the Association for Assessment and Accreditation of Laboratory Animal Care International (AAALAC). All the animal experiments were carried out in Beijing Center for Drug Safety Evaluation (Permit Number: 2012-17), in accordance with a protocol approved by the Institutional Animal Care and Use Committee of Beijing Institute of Pharmacology and Toxicology. The Balb/c nude mice (male, 6–8 weeks, obtained from the Institute of Laboratory Animal Science. CAMS and PUMC, China), bearing subcutaneous H1299 xenograft approximately 200–300 mm^3^ in size, were injected intraperitoneally (i.p) with different p53 fusion proteins (5 mg/kg) everyday. Saline buffer-treated mice were served as the control. After 12 days, mice were narcotized with CO_2_ and then perfused with phosphate-buffered saline through the heart to wash the blood out of tissues. The tumor tissues were harvested and measured the weights and diameters to calculate the inhibition rate of tumors growth. The plasma biochemical parameters of mice were also determined. The liver and the tumor lobule were fixed with absolute methanol for 1 week, and embedded in paraffin. Sections were cut at 3 microns, and placed on charged slides. Then paraffin sections were prepared and analyzed by immunohistochemistry using HistostainTM-Plus Kits (Zymed, America). An avidin/biotin horseradish peroxidase system was used with DAB to localize the site of p53 protein. Finally, sections were counterstained in hematoxylin, mounted and photographed. 

## Results

### The expression of p53, GnRH-p53 and GnRH III p53 fusion proteins

**Figure 1:**
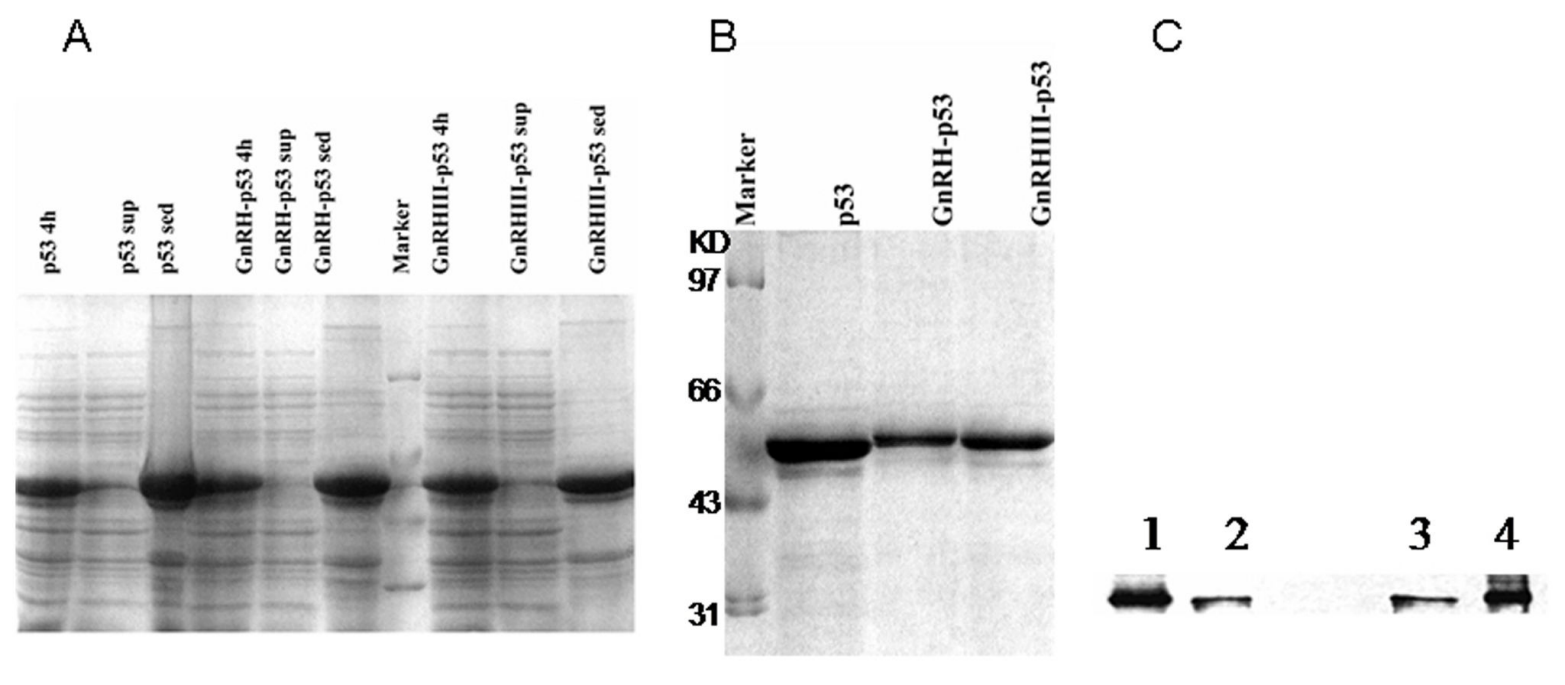
The expression and purification of GnRH-p53 and GnRH III-p53. (A) The expression of GnRH-p53 and GnRH III-p53 in *E.coli* analized by SDS-PAGE(12%). Marker, marker proteins, Marker: 97.4, 66.2, 43.0, 31.0kDa; Commassie staining. (B) Proteins after renature analyzed by SDS-PAGE (12%). M, Marker proteins; Commassie staining. (C)Western blot of p53, GnRH-p53 and GnRH III-p53. Mouse anti-p53 monoclonal antibody was used; the slices were visualized with ECL. 1, GnRH-p53; 2, p53; 3, p53; 4,GnRH III-p53.

Full length human wild-type p53 cDNA , GnRH and GnRH III genes were combined and cloned into the pET28a vector to construct the fusion protein expressing vector. Ten amino acids sequences of GnRH or GnRH III in the fusion proteins were added before that of p53. The GnRH-p53, GnRH III-p53 and p53 proteins were expressed in *E. coli*., and purified respectively (Figure 1A). The purified proteins had a high purity and were visualized as single bands migrating at 53, 54 and 54 kDa on SDS-polyacrylamide gel electrophoresis (Figure 1B). Mouse anti-p53 monoclonal antibody was used in the detection of Western blotting; the slices were visualized with ECL. All of fusion proteins could be recognized by anti-p53 antibody DO-1 (Figure 1C).

### Cellular Growth Inhibition Induced by GnRH-p53 and GnRH III-p53

**Figure 2:**
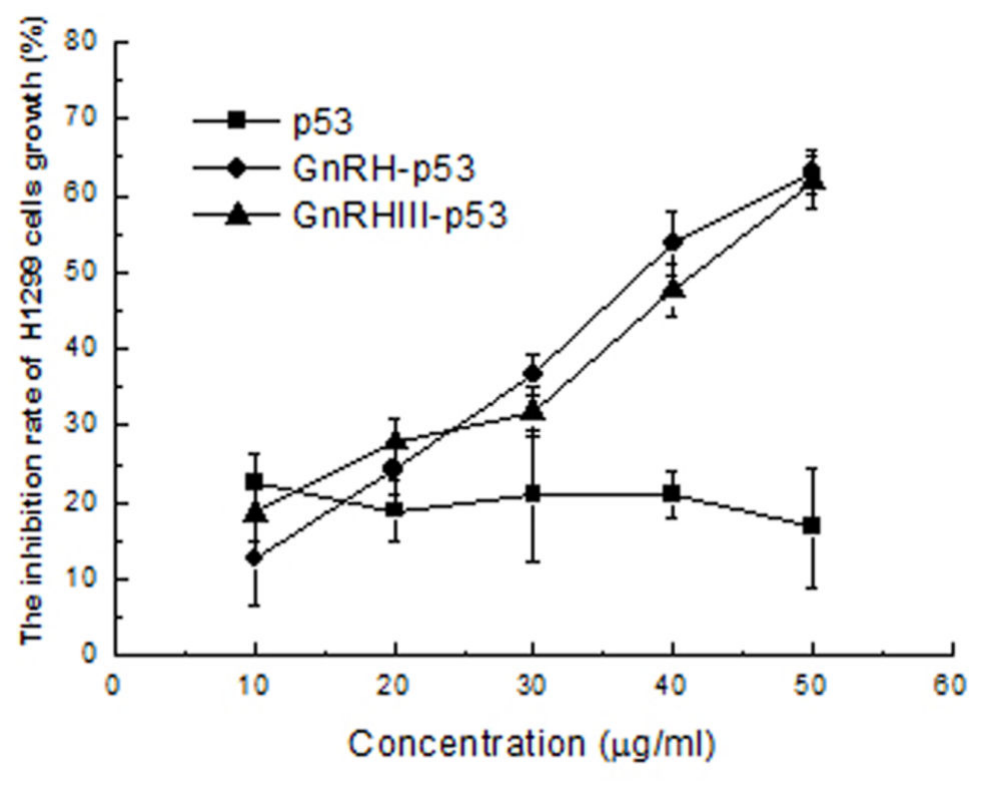
Inhibition effects of H1299 cells growth induced by p53 fusion proteins. Cell viability was assessed with MTT assay. The values of optical density (OD) were measured at 540 nm. The inhibition rate(%)=(OD_control_-OD_sample_)/ OD_control_ ×100.The results are displayed as mean ± S.D. Each assay was performed in quadruplicate.

**Figure 3:**
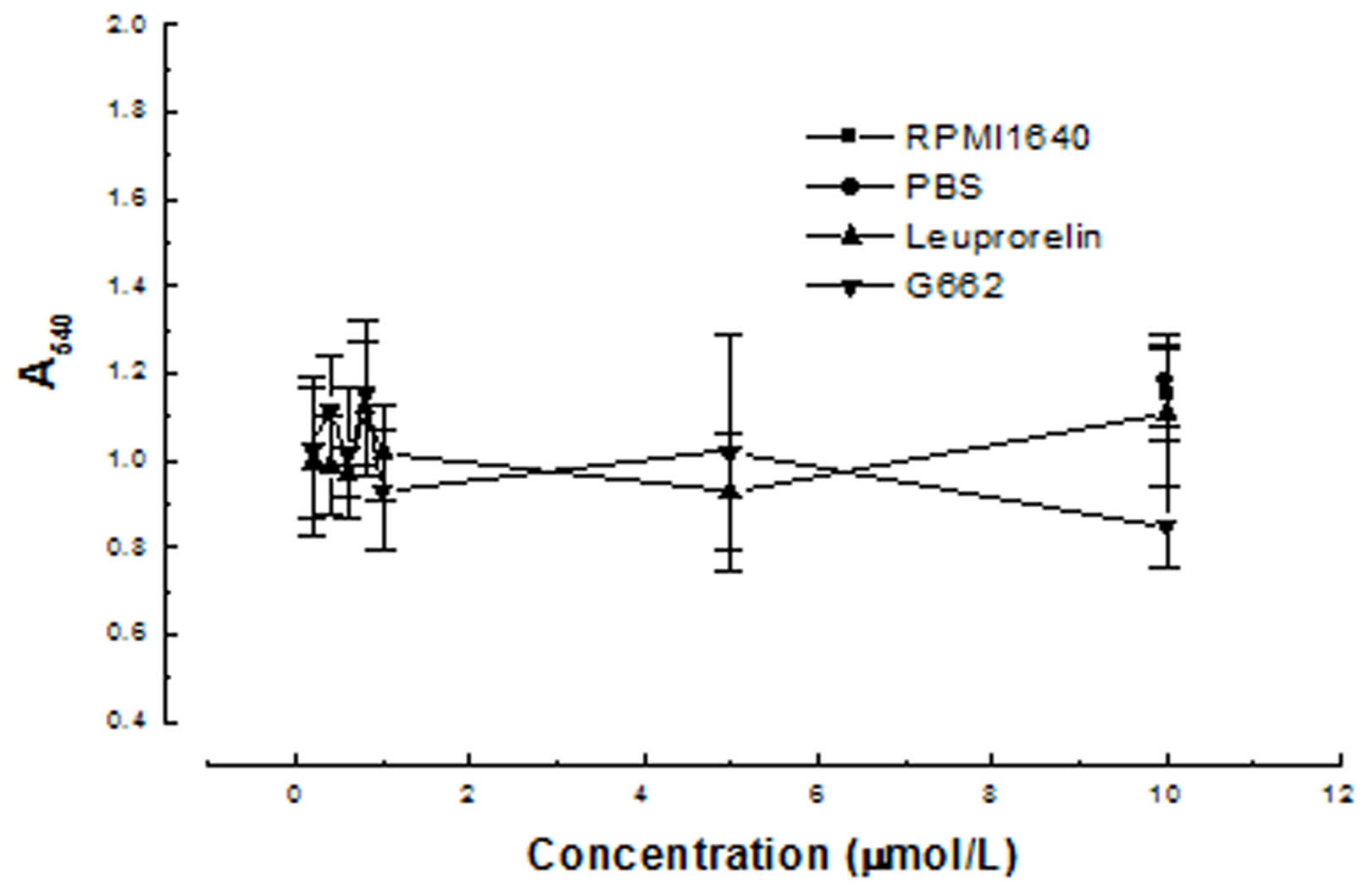
Inhibition effects of cellular growth induced by GnRH analogues. Cell viability was assessed with MTT assay. The values of optical density (OD) were measured at 540 nm. The results are displayed as mean ± SD. Each assay was performed in quadruplicate. Leuprorelin, GnRH-R agonist; G662, GnRH-R antagonist.

MTT method was used to determine the viability of H1299 treated with p53 fusion proteins. GnRH analogues, Leuprorelin and G662 were used as positive controls. The result in Figure 2 demonstrated that p53 did not inhibit the viability of tumor cells and its fusion proteins, GnRH-p53 and GnRH III-p53 exhibited their high inhibition abilities on tumor cell growth. Compared with the result from Leuprorelin and G662, GnRH-p53 and GnRH III-p53 exerted stronger tumor cytotoxicity at the same molar concentrations (Figure 3).

### The permeability of GnRH p53 and GnRH III-p53 into tumor cells

**Figure 4:**
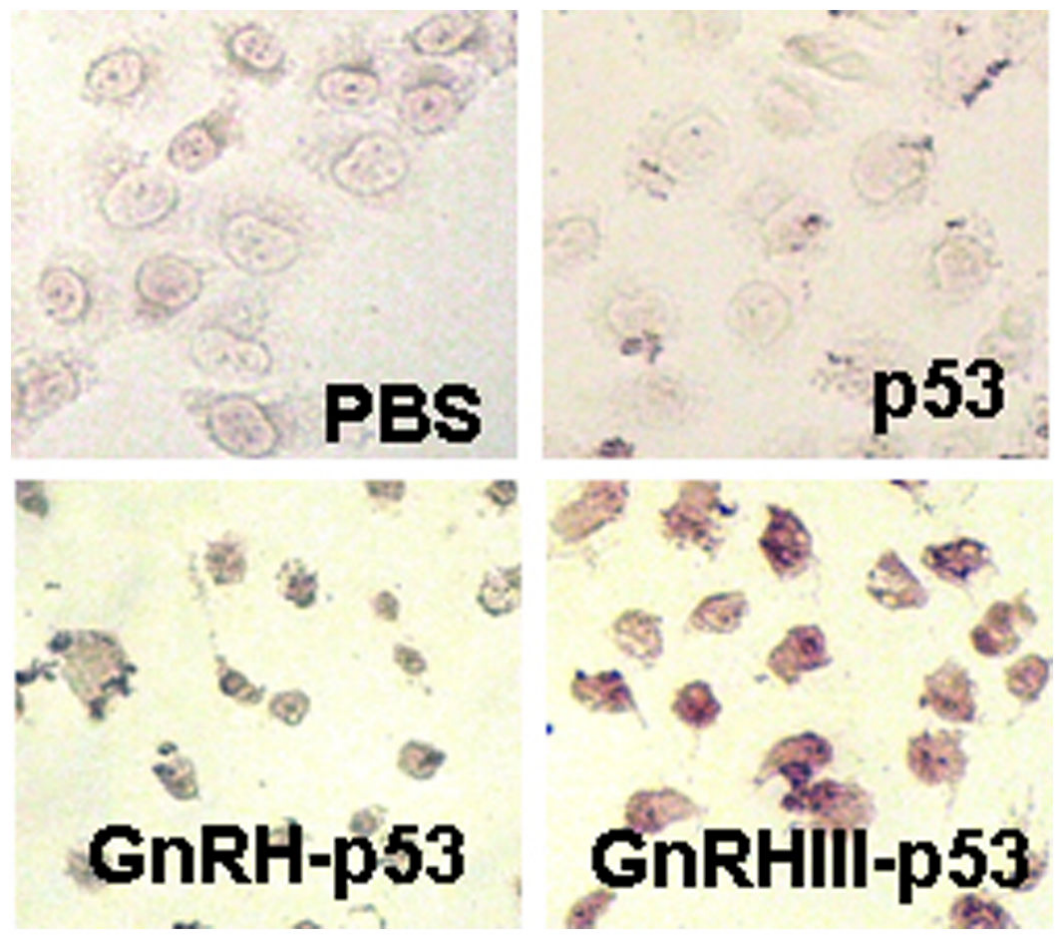
Immunocytochemistry analysis of p53 proteins in H1299 cell (200×). Immunocytochemical assay was performed using biotin-streptavidin-HRP method with monoclonal mouse anti-p53 antibody and visualized with DAB.

**Figure 5:**
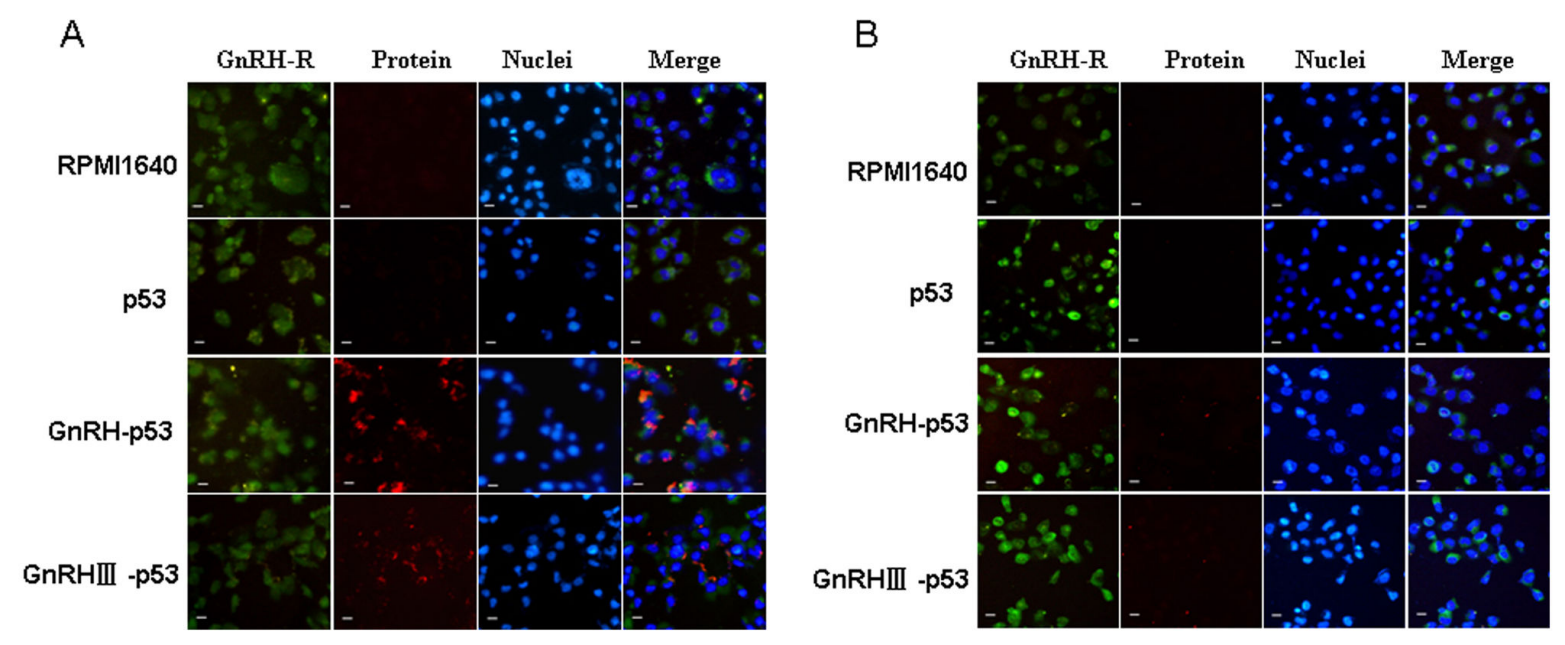
Cell permeation of fusion protein by immunofluorescence analysis. **(200×, scale bar=20 μm)**. A, H1299 cells were incubated at 37°C with 40 μg/ml of fusion protein for 4 h; B, H1299 cells were incubated at 4°C with 40 μg/ml of fusion protein for 4 h. The green fluorescence (FITC) marked the GnRH receotor in H1299 cells membrane and the red fluorescence (TRITC) showed the fusion proteins; Hoechst 33342 dyed the cell nuclei.

To measure whether p53 protein could be delivered into cells by fusion with GnRH or GnRH III, H1299 cells were treated with p53, GnRH-p53 and GnRH III-p53, respectively. Immunocytochemistry analysis and immunofluorescence analysis were used to trace intra-cellular delivery of p53. The result showed that the p53 fusion proteins conjugated with GnRH and GnRH III could permeate into the H1299 cells and were located in the cytoplasm and nucleus (Figure 4 and Figure 5A). It was interested that both GnRH-p53 and GnRH III-p53 is unvisible when cells were treated at 4°C (Figure 5B). The permeability of p53 fusion proteins might depend on the GnRH-R mediated endocytosis because both fusion proteins could not pass through H1299 cell membrane at 4°C.

### Apoptosis in H1299 induced by GnRH-p53 and GnRH III-p53

Wild-type p53 is one pivotal tumor suppressor relied on its transcriptional activity to up-regulate downstream genes especially associated with cell apoptosis. To assay the p53 activity induced by GnRH-p53 and GnRH III-p53, Flow cytometry assay was used. 

**Figure 6:**
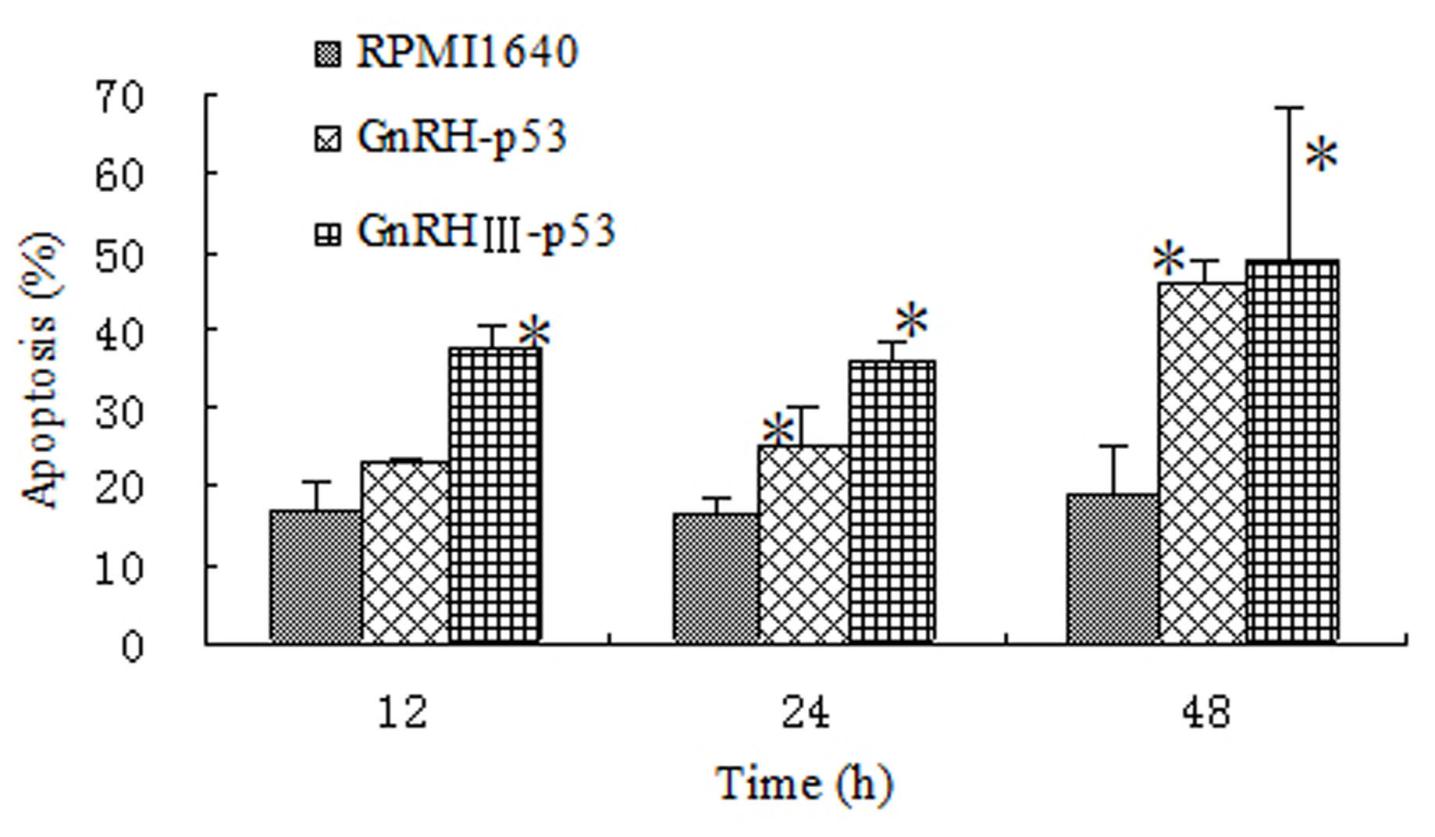
Effects of fusion protein induced apoptosis in H1299 cell by flow cytometer. The PI and FITC-AnnexinV positive cells were apoptosis cells. Data were expressed as mean±S.D. (n=3). *p<0.05, compared with RPMI1640 control; **p<0.01, compared with RPMI1640 control.

The results showed that the percentage of apoptosis cell increased with the exposure time of the proteins. The control cells were PI negative. Dead cells were brightly PI positive. FITC-AnnexinV staining was used to distinguish the cells died from apoptosis with those died from necrosis. The result showed that p53 fusion proteins could induce early apoptosis in H1299 cells (Figure 6). 

### Antitumor effect of GnRH-p53 and GnRH III-p53 in vivo

**Figure 7:**
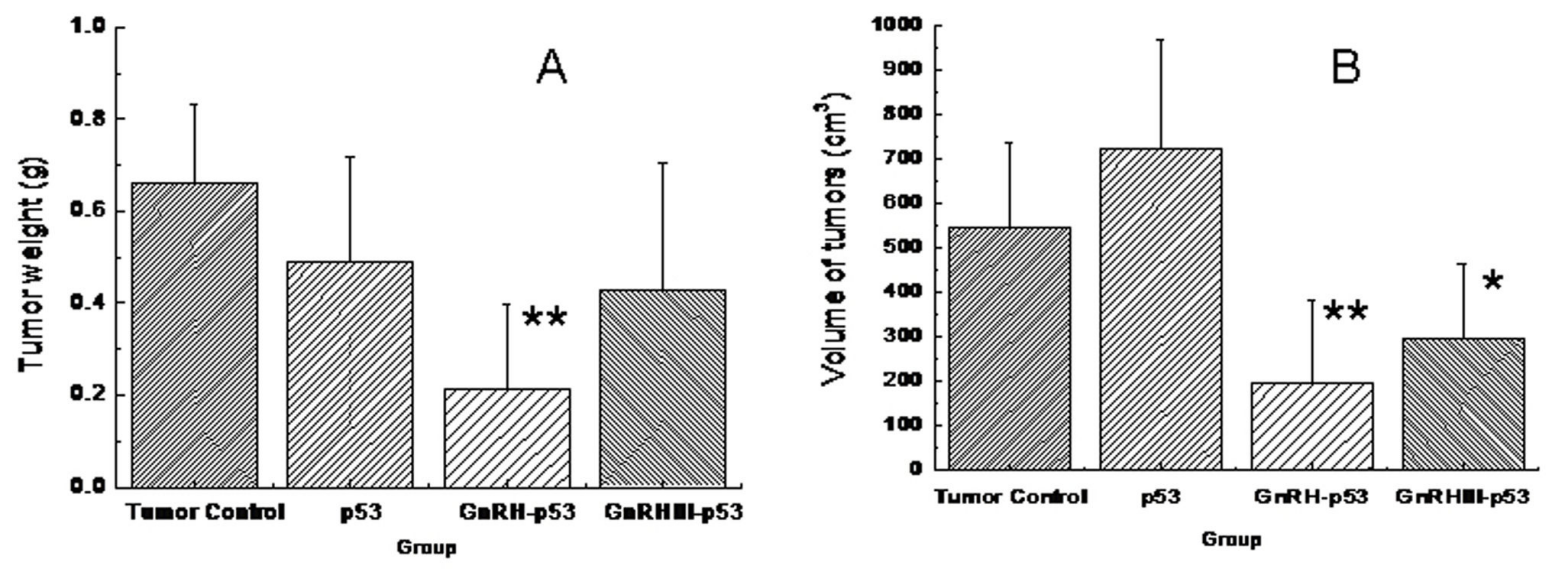
Inhibition of tumor growth induced by p53 fusion proteins. A, the tumor weight; B, the volume of tumors; V= (LW^2^)/2, L is the long axis and W is the short axis of the tumor. Data were expressed as mean±S.D. (n=6). *p<0.05, compared with tumor control (TC); **p<0.01, compared with TC.

**Figure 8:**
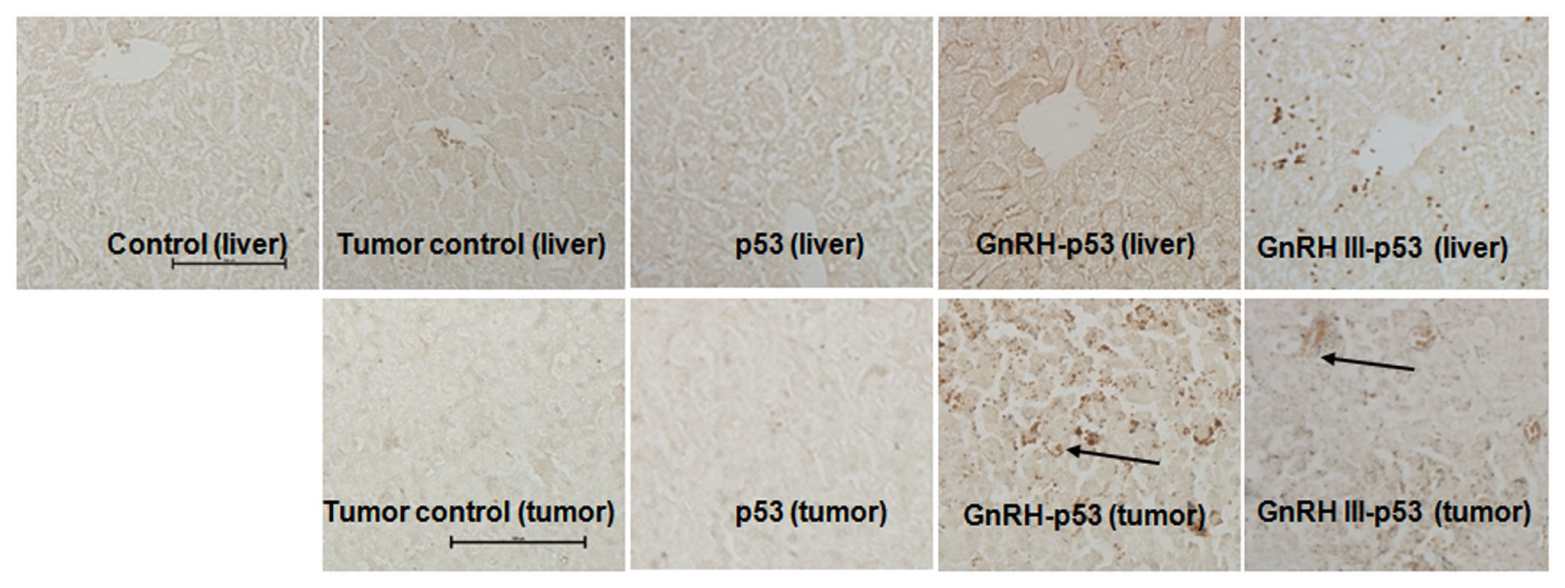
The selective stabilization of p53 fusion proteins in liver tissues and tumor tissues of mice bearing tumors. Immunohistochemistry assay was performed using biotin-streptavidin-HRP method with mouse anti-p53 monoclonal antibody (DO-1) and visualized with DAB. Original magnification 200×. Scale bar = 100 μm.

**Figure 9:**
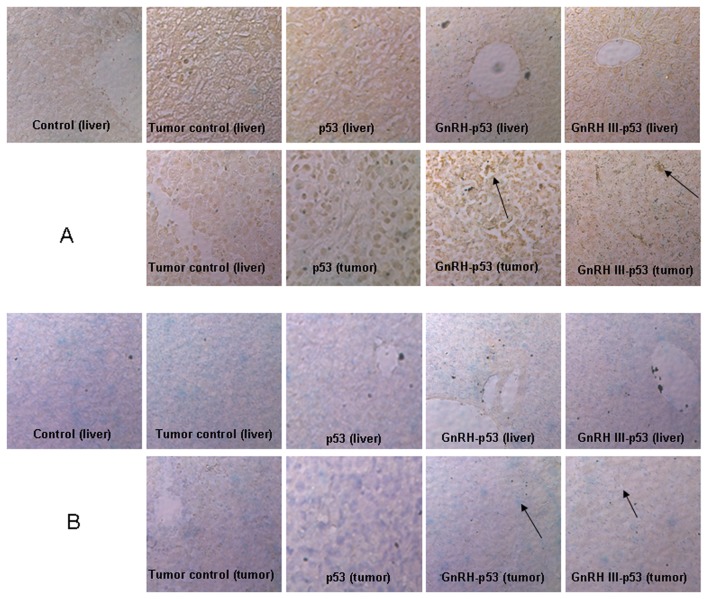
Immunohistochemistry analysis of Caspase-3 protein and p21 protein in liver tissues and tumor tissues of mice bearing tumors. Immunohistochemistry assay was performed using biotin-streptavidin-HRP method with rat anti-Caspase-3 monoclonal antibody and mouse anti-p21 monoclonal antibody, and visualized with DAB. Original magnification 200×. A, Caspase-3 protein; B, p21 protein.

Balb/c nude mice bearing subcutaneous H1299 cells xenograft were daily treated i.p. with p53 fusion proteins at the dosage of 5 mg/kg and saline buffer. The tumor growth was monitored over a 12 days period. As displayed in Figure 7, both mass and volume of tumor form mice treated with GnRH-p53 or GnRH III-p53 were lower than that in control mice treated with saline (GnRH-p53, p<0.01; GnRH III-p53, p<0.05). We also analyzed the potential antitumor mechanism of these fusion proteins. The p53 associated gene expression was assayed by immunohistochemistry. The result indicated that GnRH-p53 and GnRH III-p53 aggregated in the tumor tissues (Figure 8) and they could induce the expression of caspase-3 and p21 proteins in tumors (Figure 9). Furthermore, to illustrate the potential side effect of these two fusion proteins in vivo, the plasma biochemical parameters of mice were measured. The result indicated that there were no obvious side effects observed in mice treated with GnRH-p53 and GnRH III-p53 (Tab. 2). 

**Table 2:** The values of biochemical indicator in plasma of mice bearing tumors.

Parameters	Group				
	Control	Tumor control	p53	GnRH-p53	GnRH III-p53
ALT (U/L)	42.83±4.54	55.50±16.66	45.14±4.66	43.00±8.58	49.17±13.15
AST (U/L)	115.17±18.39	119.00±12.05	123.63±25.24	115.33±11.54	115.50±15.62
TP (g/L)	53.12±1.76	53.42±2.59	54.55±1.67	53.78±1.92	52.77±0.91
ALB (g/L)	29.63±0.82	28.88±0.91	28.87±0.52	27.72±0.78**^+^	27.15±0.69**^++^
UREA(mmol/L )	7.45±1.06	7.28±0.95	7.09±0.46	7.25±0.77	6.87±0.95
GLU (mmol/L)	9.53±0.62	8.70±0.36	9.12±1.35	9.08±0.670	9.05±0.98
CRE (μmol/L)	49.00±3.46	50.33±2.58	51.13±1.51	49.67±1.63	47.17±2.23*^+^
CHO (mmol/L)	1.98±0.08	2.10±0.17	1.95±0.06	1.96±0.09	1.93±0.12*
TG (mmol/L)	1.43±0.16	1.36±0.31	1.63±0.31	1.34±0.31	1.12±0.22*^++^

Data were expressed as mean±S.D. (n=6). *p<0.05, compared with tumor control (TC); **p<0.01, compared with TC. ^+^ *p*<0.05, compared with p53; ^++^ *p*<0.01, compared with p53.

## Discussion

Cell canceration and dropout of growth caused by genetic deletion and mutation are the main mechanisms during tumorigenesis. As a tumor suppressor protein, p53 is intimate associated with human malignant tumor. Epidemiological data demonstrated that p53 is mutated in more than half of human tumors and most of the mutations in p53 are located in the DNA-binding core domain. Delivering p53 into tumor cells could restore p53 function and inhibit tumor growth. However, the utility of wild type p53 is limited because of its poor permeability and low targeting. In this study we constructed GnRH-p53 and GnRH III-p53 fusion proteins for antitumor therapy by targeted delivering them into GnRH-R positive cancer cells though receptor-mediated endocytosis.

Tumor-targeting peptides are intensively investigated candidates for the specific delivery of anticancer drugs. It was found that receptors for peptide hormones such as GnRH and somatostatin are more intensively expressed on cancer cells, compared to normal cells and serve as targets fore-peptide ligands to cytotoxic drugs [21,22]. People found that GnRH-R were expressed in cell membrane of tumors derived from the breast, prostate, colon and liver. GnRH III has been shown that it has lower endocrine effect in mammals than the human GnRH (GnRH-I, also called LH–RH), it binds to the GnRH receptors on cancer cells and exhibits anti-proliferative effect on many types of GnRH receptor-positive tumors. lGnRH-III contains only L-amino acids, which are presumable to avoid undesirable edematogenic activity [23]. These data indicate that the therapeutic use of lGnRH-III has lower risk of complications than the application of GnRH analogs. The anticancer drug daunorubicin was attached via oxime bond to the GnRH-III as a targeting moiety [24]. GnRH-III has weaker endocrine activity compared with GnRH but it can inhibit the growth of GnRH-R positive human cancer cells. Therefore GnRH-III might have a stronger targeting anti-tumor effect than GnRH in human tumors.

In this study, GnRH and GnRH III were used to deliver p53 into H1299 cells which were p53-deficient and had high GnRH-R expression on the cell membrane (Figure 4 and Figure 5A). The permeability of p53 fusion proteins might depend on the GnRH-R mediated endocytosis because both fusion proteins could not pass through H1299 cell membrane at 4°C(Figure 5B)[25]. After GnRH or GnRH III introduced fusion proteins into cells, p53 protein inhibited H1299 cells growth in vitro. In addition, our study showed that GnRH-p53 significantly inhibited the proliferation of SH-SY5Y cells, but it had less effect on the growth of SW480 cells. However, GnRH III-p53 had high inhibition effect on the proliferation of SW480 cells and less effect on SH-SY5Y cells (Figure S1). These results indicated that the main inhibition ability of the p53 fusion protein on cell growth was related to the cell lines, not much to GnRH or GnRH III and the exogenous protein introduction. We also constructed GnRH-EGFP and EGFP gene which cloned into the pET28a vector. The EGFP gene was sourced from pEGFP-N2 plasmid (GenBand Accession: U57608). The GnRH-EGFP and EGFP proteins were expressed as solubility and purified by affinity chromatography. MTT method was used to determine the viability of H1299 cells treated with GnRH-EGFP and EGFP fusion proteins. The results showed that GnRH-EGFP exhibited weaker inhibition ability on H1299 cells growth compared with GnRH-p53 and presented very significant difference when the same molar concentrations of proteins were added (Figure S2). As indicated above, the inhibition activity of fusion proteins was mainly depended on the p53 protein. The intracellular delivery of p53 fusion proteins exerted the reduction of tumor volume and tumor weight in vivo (Figure 7). Their anti-tumor effect was functioned by the apoptosis and cell cycle arrest induced by p53 (Figure 6 and Figure 9). The distribution of GnRH-p53 in bearing cancer mice tumor tissues was more widespread than GnRH III-p53 within the same injection dose (Figure 8), which resulted in the different of tumor inhibition activity.

Our studies in vivo and in vitro showed that GnRH and GnRH III, as ligands of GnRH-R, could recognize the receptor expressed on H1299 cells and then delivered p53 fusion proteins into tumor cells and then exerted their targeted antitumor functions with limited side effects. These fusion proteins might be new biological agents on targeting tumor therapy.

## Supporting Information

Figure S1 **Inhibition effects of cells growth induced by p53 fusion proteins.** Cell viability was assessed with MTT assay. The values of optical density (OD) were measured at 540 nm. The inhibition rate(%)=(OD_control_-OD_sample_)/ OD_control_ ×100.The results are displayed as mean ± S.D. Each assay was performed in quadruplicate.
(TIF)

Figure S2 **The inhibition of cells growth induced by EGFP, p53 and their GnRH fusion proteins.** H1299 cells were respectively treated with different proteins at the final concentration of 0.9 μM for 48h (p53 and GnRH-p53) or 72h (EGFP and GnRH-EGFP). Cell viability was assessed with MTT assay. The results are displayed as mean ± S.D. Each assay was performed in quadruplicate. **p<0.01, compared with EGFP, GnRH-EGFP and p53 treatment respectively.
(TIF)
