# Supplementary figures and images for: The Novel Fusion Proteins, GnRH-p53 and GnRHIII-p53, Expression and Their Anti-Tumor Effect
Source: PLoS One. 2013 Nov 4;8(11):e79384. doi: 10.1371/journal.pone.0079384 (PMC3817058; doi:10.1371/journal.pone.0079384)

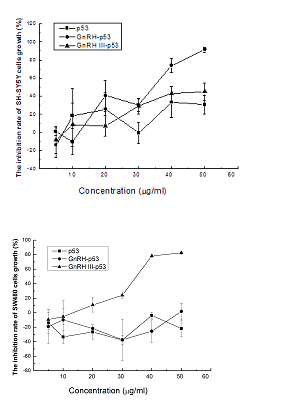

Supplement: Figure S1 — Inhibition effects of cells growth induced by p53 fusion proteins. Cell viability was assessed with MTT assay. The values of optical density (OD) were measured at 540 nm. The inhibition rate(%)=(ODcontrol-ODsample)/ ODcontrol ×100.The results are displayed as mean ± S.D. Each assay was performed in quadruplicate. (TIF) [file pone.0079384.s001.tif]

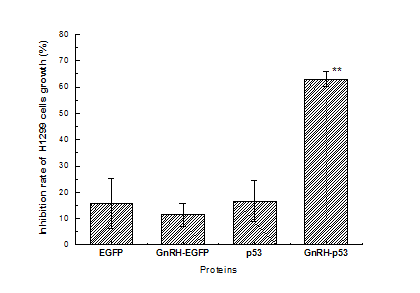

Supplement: Figure S2 — The inhibition of cells growth induced by EGFP, p53 and their GnRH fusion proteins. H1299 cells were respectively treated with different proteins at the final concentration of 0.9 μM for 48h (p53 and GnRH-p53) or 72h (EGFP and GnRH-EGFP). Cell viability was assessed with MTT assay. The results are displayed as mean ± S.D. Each assay was performed in quadruplicate. **p<0.01, compared with EGFP, GnRH-EGFP and p53 treatment respectively. (TIF) [file pone.0079384.s002.tif]
